# Bone Grafts in Trauma and Orthopaedics

**DOI:** 10.7759/cureus.17705

**Published:** 2021-09-04

**Authors:** Maheswaran W Archunan, Sandris Petronis

**Affiliations:** 1 Trauma and Orthopaedics, Norfolk and Norwich University Hospital, Norwich, GBR; 2 Trauma and Orthopaedics, Rīga Stradiņš University, Riga, LVA

**Keywords:** bone grafts, orthopedics and trauma, bone reconstruction, bone tissue engineering, bone injury

## Abstract

Worldwide, there are millions of patients each year suffering from bone-related illness due to trauma, degenerative diseases, infections or oncology that require orthopaedic intervention involving bone grafts.

This literature review aims to analyse the characteristics of the different bone grafts: autografts, allografts and synthetic bone substitutes. The review will assess their medical value based on their effectiveness as well as scrutinising any drawbacks. The goal is to identify which options can give the optimal result for a patient being treated for a bone defect.

Bone autografts remain the gold standard since there are no issues with histocompatibility or disease transmission while possessing the ideal characteristics: osteogenicity, osteoconductivity and osteoinductivity. However, synthetic options such as calcium phosphate ceramics are becoming popular as a viable alternative for treatment since they can be produced in desired quantitates and yield excellent results while not having the problem of donor site morbidity as seen with autografts. Furthermore, advancements in fields such as bone tissue engineering and three-dimensional printing are generating promising results and could provide a path for excellent treatment in the future. The emergence of such innovations highlights the importance and the constant need for improvement in bone grafting.

## Introduction and background

Worldwide, an estimated 2.2 million orthopaedic procedures involving bone grafting take place annually [1], with the incidence rate projected to increase by 13% each year [2]. Bone substitutes can be natural, synthetic or composite materials. The first recorded surgery involving bone grafts was in 1668 [3]. However, American Orthopaedic surgeon Fred H. Albee is credited for pioneering bone graft surgery in 1906. In 1965, Dr. Marshall Urist discovered demineralized bone matrix and bone morphogenic proteins with the first marketed demineralized bone matrix available in 1991.

Bone histology

Bone is a specialized connective tissue characterized by a mineralized extracellular matrix and cellular components, which include osteoprogenitor cells, osteoblasts, osteocytes and osteoclasts. The bone matrix consists of organic and inorganic components with the latter accounting for about 70%-75% of bone mass [4]. The osteoid is the unmineralised organic component consisting of type 1-collagen fibers and acidic ground substance made up of proteins, carbohydrates, proteoglycan aggregates and osteonectin [5]. The inorganic component is primarily composed of calcium phosphate and calcium carbonate. Crystallisation of these minerals results in the formation of hydroxyapatite. The tensile strength of bone is a result of the collagen fibers and the hydroxyapatite crystals attribute to its compressional strength [6].

Osteoprogenitor cells found in the periosteum and endosteum are essentially stem cells of mesenchymal origin that give rise to osteoblasts. Osteoblasts are metabolically active bone-forming cells. They are responsible for producing and secreting osteoid, the organic component of the extracellular matrix, which subsequently becomes calcified [7]. Once the osteoblasts become trapped in the organic matter they differentiate into osteocytes. Osteocytes are mature cells that are responsible for maintaining bone tissue by providing transport channels for nutrients and waste products via their cytoplasmic processes. Lastly, osteoclasts are multinucleated cells derived from monocytes. They are responsible for bone resorption, therefore, are essential for the repair, remodelling and growth of bone [7,8].

Ideal bone graft properties

Bone grafts properties can be divided into three groups based on their biological actions: osteogenicity, osteoinductivity and osteoconductivity. Osteogenic capabilities of the bone graft reflect on their ability to synthesize new bone. Osteoinductive grafts can stimulate mesenchymal cells and osteoprogenitor cells in the surrounding host tissue to differentiate into osteoblasts, thus, promoting the formation of new bone. Osteoconductive grafts provide passive microscopic and macroscopic scaffolding that supports bone formation and bone growth by providing a porous structure through which cells such as osteoblasts can migrate through and blood vessels can grow into, consequently incorporating the graft tissue into the host’s bone [9,10]. An ideal bone graft has all three of the aforementioned properties as well as being of low cost, lack risks for infection and being readily available in any desired quantities.

Incorporation of bone graft

Bone graft incorporation can be generalized into two phases. The first phase begins with the formation of hematoma around the implanted bone, followed by the release of cytokines and growth factors. Subsequently, inflammatory processes take place resulting in the development of fibrovascular tissue. Although, it is worth noting that the incorporation of grafts differs between cortical and cancellous bone grafts due to their structural difference. The vascular response seen with cancellous bone is much greater than cortical bone since it is much more porous.

## Review

Natural bone grafts

Autograft

Autograft bone is harvested from the patient’s own body from a different unaffected site. The graft is most often obtained from distant sites, the most popular being the posterior iliac crest since graft tissue obtained here is said to have the highest osteogenic potential and it provides both cancellous and cortical bone. Other sites include femoral greater trochanter, proximal-distal tibia, calcaneus and distal radius. Autogenous bone grafts remain the gold standard therapy because to some extent they have all three of the desired properties: osteogenic, osteoinductive and osteopromotive [9]. Since the donor and recipient are the same individuals there will be no issues with histocompatibility and there are no risks of transmitting disease. Although patients can potentially suffer from donor site morbidities such as chronic pain, hypersensitivity, paresthesia, pelvic instability and infections. The complications rate following iliac crest bone graft harvesting ranges between 2% and 36% [2].

Allograft

Allografts bone graft material is harvested from living donors or cadavers. Harvested bone tissue is processed to decrease the risk of host-versus-graft immune response and facilitates the removal of harmful substances from the bone tissue that may transmit disease. Through processing methods, the shape and size of the graft material can be altered to fit for their intended use. However, the processing step does seem to alter the structure of the graft thus affecting its mechanical competency and its ability to stimulate bone healing [11]. The graft material is preserved by deep freezing or freeze-drying. Deep frozen allografts are advantageous since they retain their structural properties. On the other hand, the advantage of freeze-drying is that it allows storage of the grafts at room temperature but freeze-dried allografts can become fragile to torsion or bending since microfractures form along the collagen fibers of the allograft, hence, deteriorating its structural strength [12]. Allografts have many advantages, namely the abundant supply of graft material, which can be obtained in the desired configuration. There is no need to compromise host structures to obtain the graft tissue, and subsequently, there will be no donor-site morbidity as seen with autografts. On the other hand, regardless of the processing and sterilization of the grafts, there will always be a risk of host immune response and disease transmission. That being said, disease transmission is extremely rare with the risk of contracting HIV or hepatitis B/C estimated to be about 1/1.6 million. 

Synthetic bone substitute 

There has been significant research and development to generate alternative options in the form of synthetic bone substitutes. The aim is to provide a cost-effective graft substitute that is similar in structure and strength of human bone, support new bone formation, bioactive, biocompatible, osteoconductive, osteoinductive, and osteointergrative.

Metal

Trabecular metal technology is an innovative 3D material made from the metal tantalum that shows excellent biocompatibility and resistance to corrosion. It is structurally similar to cancellous bone. Trabecular metal technology surfaces have nano-textured topography while exhibiting high porosity of up to 80% that are consistent in size and shape [13].

Ceramic

Ceramic graft options include calcium phosphate, calcium sulphate, calcium phosphate cement and bioactive glass. Ceramic based bone substitutes account for 60% of the synthetic graft market and have been very popular because they are bioactive providing good osteoconductivity, become integrated into the host tissue very well, can be obtained in any desired amount while there are no risks of disease transmission or donor site morbidity as seen with the natural bone grafts [14]. Drawbacks of ceramics are the relatively high cost and the fact that they are brittle with very low tensile strength. Calcium phosphate cement, which is available in an injectable form, allows the possibility of intraoperative moulding of the graft. The cement consists of a mixture of dicalcium phosphate anhydrite and tetracalcium phosphate. Once injected into the graft site using a dual-chamber syringe, the cement begins to harden and is converted to porous hydroxyapatite with osteoconductive properties. Calcium phosphate cement has been successfully used to fill bone defects but while the recipe for the cement is FDA approved it is still under research and development to be perfected.

Bioactive glass is also a ceramic-based bone substitute. It exhibits very good osteoconductive and osteointergrative properties. Once implanted, the ions in the bioactive glass such as Na+ and K+ react with the extracellular fluid, resulting in the production of a silica-rich gel layer forming over the implant which is highly porous [15]. Subsequently, Ca2+ and PO43+ from the extracellular fluid react and then precipitate onto the silicone-rich layer forming a coat of hydroxyapatite on which blood proteins, growth factors and collagen will be adsorbed. The newly formed hydroxyapatite layer is like the naturally found version in bone and attracts macrophages to initiate tissue healing as well as osteoblasts and osteoprogenitor cells to begin new bone formation. One difficulty faced with bioactive glass is that they may be difficult to fix to the bone since they can be very difficult to shape and when attempted may break in the process [16].

Polymer Based

Polymer-based bone substitutes provide a scaffold structure promoting osteoconductivity. Synthetic polymers can be manufactured in any amount as well as having their structure and composition being designed as desired. There is also no concern of immunogenicity or the presence of pathogenic agents. Non-degradable polymers such as ultra-high molecular weight polyethylene have been used in the production of acetabular cups used in total hip arthroplasty [17]. Over the past decade, degradable polymer bone substitutes have been used more and more since it is ideal to have a synthetic graft that is completely resorbed leaving no foreign material behind. However, polymers do not provide much mechanical strength, so it is best when they are used together with another bone substitute.

Coralline Hydroxyapatite

Coralline hydroxyapatite is obtained from calcium carbonate extracted from sea coral. The material is exposed to heat and pressure while in an aqueous phosphate solution, resulting in the conversion of the calcium carbonate exoskeleton to calcium phosphate [16]. Coralline hydroxyapatite has high compressional strength but does not offer much tensile strength like synthetic hydroxyapatite. It is generally accepted that the minimum requirement for the pore size in biomaterials for promoting cell migration and vascular ingrowth is 45-100μm [16], The problem with synthetic hydroxyapatite is that the pore size is not consistent, which ultimately affects its effectiveness as a bone graft. In contrast to this, the structure of coralline hydroxyapatite is very similar to cancellous bone and the naturally formed pores tend to have a more constant size. The commercially available products can have a mean pore size of 200μm or 500μm [9]. Coralline hydroxyapatite has been successful in treating metaphyseal defects such as tibial plateau fracture. In recent times, there have been studies on animals where coralline hydroxyapatite was used as a carrier for bone morphogenic proteins with promising results being achieved [9].

Clinical use of bone grafts

Trauma

In Europe, 5.7 million patients are admitted every year due to trauma, costing health care services €78 billion each year [18]. In the UK alone, there were 186,000 bone and joint-related emergencies between 2012 and 2013 [18]. There can be cases when there is significant bone loss overwhelming the bone’s capabilities to heal, thus resulting in non-unions or mal-union. This warrants the use of bone grafts to bridge the gap between the fracture ends and promote bone healing. 

Oncology

Primary malignant bone cancer is rare, with about only 559 cases of all sarcoma subtypes in 2011 in the UK, accounting for 0.2% of all cancers [19]. Secondary bone cancers, however, are much more common and arise due to metastasis spread from other organs, most commonly from the thyroid, breast, lungs, kidney and prostate. When surgically resected, large defects are left in the treated bone, thereby, warranting the use of bone grafts to fill the voids. 

Future of bone grafts

Bone Tissue Engineering

Bone tissue engineering aims to essentially act as a source for unlimited autogenous bone tissue. Human mesenchymal stem cells have been the subject of several studies and experiments aimed at bone tissue engineering with promising results being yielded. The patient’s bone marrow is used as the source for the human mesenchymal stem cells, which are multipotent undifferentiated cells that are capable of undergoing chondrogenesis and osteogenesis [20]. The idea is to use a polymer scaffold onto which these cells grow onto while in the presence of bone stimulating factors.

3D Printing

3D printing allows the production of custom-made implants unique to a patient’s bone defect as well as ensuring that the printed product has the desired structure and composition to mimic bone architecture. The uniformity and size of pores can be carefully controlled in order to produce a complex implant that offers a scaffold with prime osteoconductivity. The implant can be printed layer-by-layer using modified hydroxyapatite powder as the feed material together with a polymer-based binder [21]. Once the printing is complete, the product is dried, cleaned and sintered at 1,250°C for two hours [22]. Exposure to the high temperature decomposes the polymer-based binder, leaving behind only the ceramic body. The aim is to have 3D printed implants clinically available in the next 10 to 20 years [22]. The problem with the high temperature used in the manufacturing process is that it makes it impossible to infuse additional agents such as bone growth factors or antibiotics. Moreover, there is the possibility of coating the 3D printed scaffolds with human stems cells harvested from the patient. This would significantly enhance the integration of the graft into the host tissue. 

Gene Therapy

Gene therapy is a promising method to promote new bone growth. The idea is to transfer genetic encoding information to the target site and induce bone healing by manipulating the endogenous host cells to produce specific proteins such as growth factors. The use of virus vectors for the expression of bone morphogenic proteins for bone formation has been successfully demonstrated in vitro and in vivo animal models [23]. Unfortunately, there are limitations to gene therapy that inhibits progress being made in this field. Firstly, there is always a risk of immune response when introducing a viral vector into the host tissue and such response can drastically deteriorate the effectiveness of the gene therapy. The use of viral vectors also raises the risk of infections. The possibility of tumour development is present if the transferred gene is encoded into a wrong position in the host’s DNA. Furthermore, for gene therapy to be successful the right gene should be targeted in the right cells but there is potential for the viral vector to target unwanted host cells.

## Conclusions

The prevalence of medical conditions related to bone or joint impairment is on the rise primarily due to the ageing population and the major impact they have on the lives of the affected individual is apparent.

Despite the presence of several choices, autografts remain the ‘gold standard’. However, synthetic bone substitutes are gradually gaining momentum in Orthopaedics, especially calcium phosphate ceramics.

The future holds tremendous promise because of current researches, especially in 3D printing and bone tissue engineering, which aim to provide grafts that have all the ideal characteristics and being available in unlimited amounts. For now, these innovations may be some years away from human trials and may not even be cost-effective initially. However, with the continuous advancement in technology, there are plenty of reasons to be optimistic. The fact that several studies such as these are taking place further emphasizes the importance of bone tissue regeneration in Orthopaedics and the constant need to improve the bone grafting options being available for treating patients.
